# Anti-Apoptosis and Anti-Fibrosis Effects of *Eriobotrya Japonica* in Spontaneously Hypertensive Rat Hearts

**DOI:** 10.3390/ijms19061638

**Published:** 2018-05-31

**Authors:** Jui-Ting Chiang, Khan Farheen Badrealam, Marthandam Asokan Shibu, Sue-Fei Cheng, Chia-Yao Shen, Chih-Feng Chang, Yueh-Min Lin, Vijaya Padma Viswanadha, Shih-Chieh Liao, Chih-Yang Huang

**Affiliations:** 1 Graduate Institute of Aging Medicine, China Medical University, Taichung 40402, Taiwan; daisy2@ms14.hinet.net; 2 Graduate Institute of Basic Medical Science, China Medical University, Taichung 40402, Taiwan; farheenkbiotech@gmail.com (K.F.B.); shibu@mail.cmu.edu.tw (M.A.S.); 3 Department of Pharmacy, Taiwan Adventist Hospital, Taipei 10556, Taiwan; 149374@tahsda.org.tw; 4 Mackay Junior College of Medicine, Nursing, and Management, New Taipei City 11260, Taiwan; 5 Department of Nursing, Mei Ho University, 23 Pingguang Road, Pingtung 91202, Taiwan; x00003061@meiho.edu.tw; 6 Department of Internal Medicine, Division of Cardiology, Taichung Armed Forces Taichung General Hospital, Taichung 40402, Taiwan; doc98049@yahoo.com.tw; 7 Department of Pathology, Changhua Christian Hospital, Changhua 500, Taiwan; yuemin0607@yahoo.com.tw; 8 Department of Biotechnology, Bharathiar University, Coimbatore 641 046, India; padma.vijaya@gmail.com; 9 School of Medicine, College of Medicine, China Medical University, 91 Hsueh-Shih Road, Taichung 40402, Taiwan; liao@mail.cmu.edu.tw; 10 Graduate Institute of Chinese Medical Science, China Medical University, 91 Hsueh-Shih Road, Taichung 40402, Taiwan; 11 Department of Biological Science, Asia University, Taichung 40402, Taiwan

**Keywords:** *Eriobotrya japonica*, SHRs, apoptosis, fibrosis

## Abstract

Myocardial apoptosis and fibrosis represent important contributing factors for development of hypertension-induced heart failure. The present study aims to investigate the potential effects of *Eriobotrya japonica* leaf extract (EJLE) against hypertension-induced cardiac apoptosis and fibrosis in spontaneously hypertensive rats (SHRs). Twelve-week-old male rats were randomly divided into four different groups; control Wistar Kyoto (WKY) rats, hypertensive SHR rats, SHR rats treated with a low dose (100 mg/kg body weight) of EJLE and SHR rats treated with a high dose (300 mg/kg body weight) of EJLE. Animals were acclimatized for 4 weeks and thereafter were gastric fed for 8 weeks with two doses of EJLE per week. The rats were then euthanized following cardiac functional analysis by echocardiography. The cardiac tissue sections were examined by Terminal Deoxynucleotidyl Transferase-Mediated Deoxyuridine Triphosphate (dUTP) Nick End-Labeling (TUNEL) assay, histological staining and Western blotting to assess the cardio-protective effects of EJ in SHR animals. Echocardiographic measurements provided convincing evidence to support the ability of EJ to ameliorate crucial cardiac functional characteristics. Furthermore, our results reveal that supplementation of EJLE effectively attenuated cardiac apoptosis and fibrosis and also enhanced cell survival in hypertensive SHR hearts. Thus, the present study concludes that EJLE potentially provides cardio-protective effects against hypertension-induced cardiac apoptosis and fibrosis in SHR animals.

## 1. Introduction

The occurrence of hypertension is increasing rapidly; as a consequence, heart failure (HF) resulting from hypertension has emerged to be a major public health concern [1]. Maladaptive cardiac remodeling has been much studied in relation to hypertension-induced HF. Cardiac remodeling occurs due to numerous complex changes in the myocardium and affects their behavior in a multifaceted manner. The consequences that precede these changes are also vast and varied. Over the years, various rationales have been put forward to explicate the mechanism governing maladaptive cardiac remodeling and HF [2]. The stimulation of neurohormonal and mechanotransduction axis are important forces that leads to cardiac remodeling preceding HF. Myocardial apoptosis, fibrosis and contractile dysfunction has been implicated as contributing factors for the maladaptive cardiac remodeling process [2]. Our previous work has endeavored influential role of Renin-Angiotensin axis against diverse cardiomyopathies including cardiac apoptosis and fibrosis [3,4,5].

Myocardial apoptosis is considered as a predictor of the unfavorable consequences in patients with failing hearts [6]. Increased levels of cardiomyocyte apoptosis have been implicated in the hypertrophied left ventricle (LV) of SHR animals and hypertensive patients [7]. Apoptosis is a well-coordinated event that involves participation of series of molecular events that eventually leads to cell death. The apoptotic pathways are controlled through the coordinated interaction of various pro-apoptotic and anti-apoptotic proteins; wherein the Bcl-2 family proteins and caspases are the main regulatory mediators. The fate of the cells towards survival or death lineage seemingly depends on the delicate equilibrium between Bcl-2 family proteins [8]. The Bcl-2 protein family consists of a vast array of pro-apoptotic and anti-apoptotic proteins. Bcl-2 is anti-apoptotic or pro-survival whereas Bad is pro-apoptotic. In essence, the pro-apoptotic protein Bad leads to disruption of mitochondrial membrane potential and release of apoptotic factors such as cytochrome c to cytoplasm. The release of these factors subsequently initiates a cascade of events eventually leading to apoptosis. The regulatory Bcl-2 protein family results in the activation of caspases which are cysteine-dependent aspartate-directed proteases. Among various caspases, cleaved caspase3 (c-Cas3) is the ultimate player that play crucial role in the execution of the apoptotic event [9].

The Akt signaling pathway is a prominent intracellular signal transduction pathway that regulates cellular survival and functions [10]. Akt consists of three isoforms i.e., Akt1, Akt2 and Akt3 that are closely related to each other [11]. Amongst the three isoforms, Akt1 appears to be the most important; phosphorylation of Akt1 at Ser473 residue activates the Akt1 kinase and initiates the signaling cascade that regulates the expression of pro-survival mediators [12].

Cardiac fibrosis represents another important contributing factor of the maladaptive cardiac remodeling process. Basically, it attenuates myocardial compliance and leads to myocardial dysfunction. The accumulation of collagen is thought to be the main factor for cardiac fibrosis. It is evident that there is an intricate network of collagen in the myocardium. The interstitium is mainly comprised of type I and type III collagen fibers [13]. The fundamental role of these intricate networks is to reinstate from deformities, preserve the alignment of the structures, and control dispensability of the cardiomyocytes besides others. Nevertheless, the anomalous accumulation of collagen, particularly type I collagen (which are larger, stiffer and more stable), has been implicated in various cardiac ailments. Under elevated Collagen I accumulation, the resultant myocardial fibrosis will be correlated with enhanced cardiac rigidity, myocardial dysfunction, feeble contractibility, compromised coronary flow etc. Myocardial fibrosis is considered as an indicator of mortality in patients with failing myocardium [13,14,15]. Furthermore, the extracellular matrix (ECM)-disrupting molecular factors including plasminogen activators (PA) and matrix metalloproteinase have been implicated in ECM remodeling. Previous studies from our lab and from other researchers have investigated the role of tissue-type plasminogen activator (tPA) and urokinase-type plasminogen activator (uPA) in myocardial remodeling [16,17,18].

Popularity of various complementary and/or alternative medicines is emerging tremendously [19,20,21]; in fact, it could be reasonable to instigate that the field of medicine are undergoing paradigm shift from the usage of synthetic drugs to the acceptability of herbal medicines. This transformation could be due to various issues, and the supporters of these changes have proposed various reasons to substantiate this changing trend. To this end, accumulating evidences from diverse research fraternities have highlighted the cardio-protective attributes of various plant species including traditional Chinese medicine [22,23,24,25,26,27]. Efforts from our lab have highlighted the efficacy of Radix *Angelicae Sinensis*, *Codonopsis pilosula*, *Alpinate Oxyphyllae Fructus*, *Platycodon grandiflorum* against angiotensin-II induced cardiac ailments in H9c2 cardiomyoblasts [28,29,30,31].

*Eriobotrya japonica* (EJ) is a popular traditional Chinese medicine with rich medicinal values. Earlier studies have shown many beneficial health-related properties of EJ including anti-oxidant and anti-inflammatory properties, particularly of EJ leaf extract (EJLE) [32,33,34]. Thus, the current study aims to elucidate the cardio-protective effect of EJLE to attenuate hypertension-induced cardiac ailments such as apoptosis and fibrosis in spontaneously hypertensive rats (SHRs).

## 2. Results

In the present study, we elucidated the plausible effects of EJLE against cardiac apoptosis and fibrosis in SHR animals. Recently, we found that EJLE shows beneficial effects against cardiac hypertrophy in SHR animals. Reckoning with these we envisage that they may owe cardio-protective attributes against cardiac apoptosis and fibrosis in SHR animals.

### 2.1. EJLE Ameliorates Cardiac Functional Characteristics in SHR Animals

**Figure 1:**
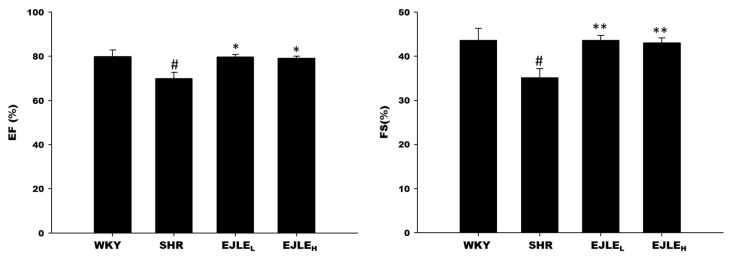
Effect of EJLE on cardiac functional characteristics of SHR animals according to echocardiographic assessment. Differences in Ejection Fraction (EF) and Fraction shortening (FS) levels determined by echocardiography in normotensive Wistar Kyoto rats (WKY), spontaneously hypertensive rats (SHRs) and SHRs supplemented with low dose (EJLE_L_) and high dose (EJLE_H_). The values are the means ± S.D. All measurements were performed post EJLE treatment. # (*p* < 0.05) indicate significant differences when compared to normotensive WKY group (SHRs vs. WKY); whereas * (*p* < 0.05) and ** (*p* < 0.01) indicate significant differences when compared to SHR group.

Echocardiographic assessment revealed that the crucial cardiac functional parameters viz. Ejection Fraction (EF) and Fraction shortening (FS) were significantly reduced in SHR group consistent with abnormal myocardium; however, EJLE supplementation in low and high dosage significantly rescued the EF and FS levels as evident from Figure 1.

### 2.2. EJLE Ameliorates Cardiac Apoptosis in SHR Heart

**Figure 2:**
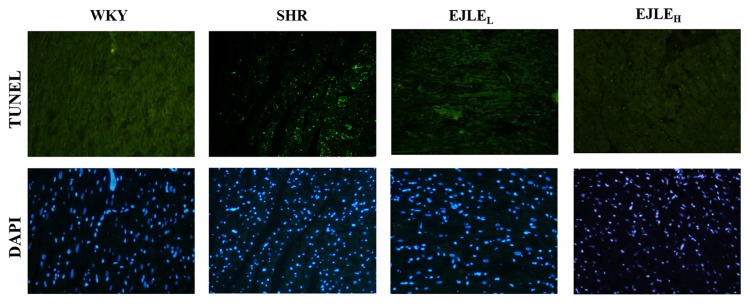
Effect of EJLE on cardiac apoptosis in SHR animals. Representative photomicrographs of TUNEL and DAPI stained nuclei in heart tissue sections of normotensive (WKY), spontaneously hypertensive rats (SHRs) and SHRs supplemented with low dose (EJLE_L_) and high dose (EJLE_H_) of EJLE. The images were acquired at 400× magnification.

Terminal Deoxynucleotidyl Transferase-Mediated Deoxyuridine Triphosphate (dUTP) Nick End-Labeling (TUNEL) and 4′,6-Diamidine-2-Phenylindole Dihydrochloride (DAPI) Staining i.e., TUNEL assay is a suitable method to detect apoptotic cells that has undergone extensive DNA fragmentation [35]. As evident from the Figure 1, the number TUNEL-positive cells stained in green were almost negligible in WKY animals; whereas numerous TUNEL-positive cells could be observed in the myocardium of SHR animals. However, EJLE supplementation showed a considerable reduction in the number of TUNEL-positive cells as compared to SHR animals (Figure 2).

### 2.3. EJLE Attenuates Apoptotic Signaling Mediators in SHR Animals

**Figure 3:**
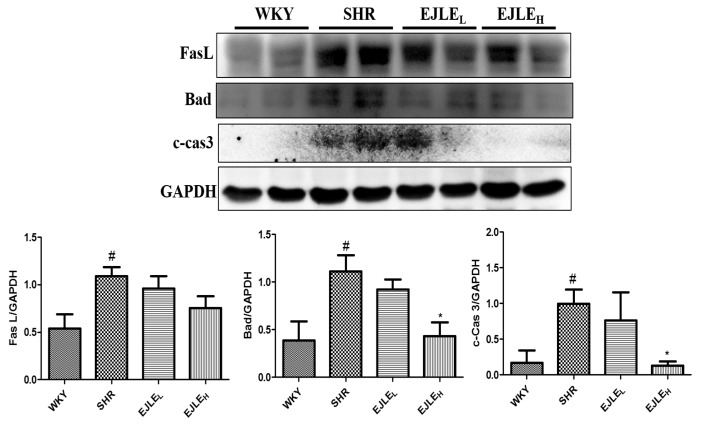
Effect of EJLE on apoptotic signaling mediators in the heart tissue sections of SHR animals. Representative Western blot depicting the changes in the levels of FasL, Bad, c-Cas3 proteins involved in apoptosis in normotensive rats (WKY), spontaneously hypertensive rats (SHRs) and SHRs supplemented with low dose (EJLE_L_) and high dose (EJLE_H_) of EJLE. Results were analyzed by one-way ANOVA using Tukey test with GraphPad Prism software (Version 5.0). # (*p* < 0.05) indicate significant differences when compared to normotensive WKY group (SHRs vs. WKY); whereas * (*p* < 0.05) indicate significant differences when compared to SHR group.

To further substantiate our findings, we analyzed crucial proteins involved in apoptosis through Western blotting. The expression of the apoptotic proteins viz. Fas L, Bad, c-Cas3 were significantly higher in SHR group when compared to those in the WKY group thereby indicating signatures of apoptosis in the heart tissue sections from SHR animals. However, expression levels of these proteins were considerably reduced by supplementation of EJLE particularly with a high dose (Figure 3).

### 2.4. EJLE Enhances Survival Markers in SHR Animals

**Figure 4:**
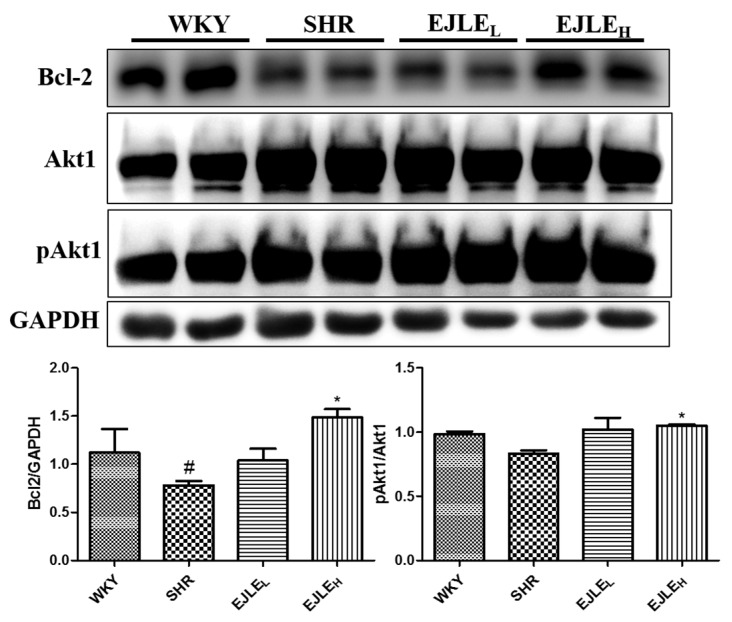
Effect of EJLE on the survival markers in the heart tissue sections of SHR animals. Representative Western blot depicting the changes in the levels of Bcl-2, Akt1, and pAkt1 proteins involved in the survival pathways in normotensive rats (WKY), spontaneously hypertensive rats (SHRs) and SHRs supplemented with low dose (EJLE_L_) and high dose (EJLE_H_). Results were analyzed by one-way ANOVA using Tukey test with GraphPad Prism software (Version 5.0). # (*p* < 0.05) indicate significant differences when compared to normotensive WKY group (SHR vs. WKY); whereas * (*p* < 0.05) indicate significant differences when compared to SHR group.

Furthermore, we have analyzed the proteins involved in the survival pathways including Bcl-2 and Akt1. Bcl-2 is a pro-survival protein and their expressions were high in control rats. On the contrary, reduced Bcl-2 expression was observed in the heart tissue sections of SHR group; however, significant increment in their expression was observed following administration of EJLE especially with high dosage. Furthermore, it could be seen that the ratio of pAkt1/Akt1 was decreased in SHR animals indicating progression toward apoptosis in these group of animals. Nevertheless, upon treatment with EJLE, this ratio was considerably shifted; owing to increment in the active form of Akt1 i.e., phosphorylated Akt1 (Figure 4). Thus, we could envisage that EJLE treatment leads to activation of pro-survival signaling cascade in SHR animals.

### 2.5. EJLE Ameliorates Fibrotic Phenomena in SHR Animals 

**Figure 5:**
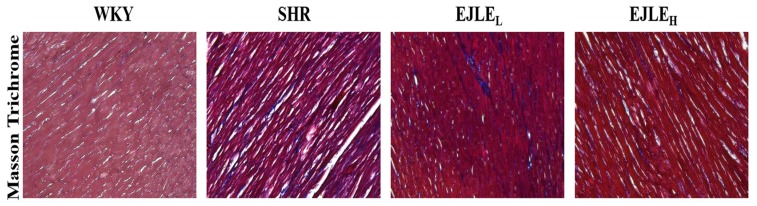
Effect of EJLE on fibrosis in the heart of SHR animals. Representative Masson’s trichrome photomicrograph depicting the effect of EJLE on the heart tissue sections of normotensive rats (WKY), spontaneously hypertensive rats (SHRs) and SHRs supplemented with low dose (EJLE_L_) and high dose (EJLE_H_) of EJLE. The heart tissue sections from all animals were processed for Masson’s trichrome staining as detailed in the materials and methods and images were acquired through microscope at 400× magnification.

As could be seen from Figure 5; LV tissue sections from WKY rats did not show any collagen accumulation; however, considerable collagen accumulation (blue trichrome stain) was evident in the LV heart tissue sections from SHR animals. Nevertheless, collagen accumulation was considerably reduced upon supplementation of EJLE, particularly under high dose regime.

### 2.6. EJLE Attenuates Fibrosis Associated Proteins in SHR Animals

**Figure 6:**
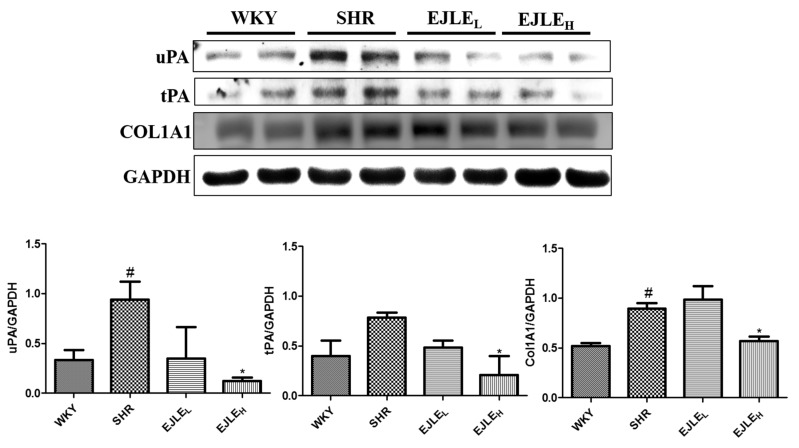
Effect of EJLE on the fibrosis associated proteins in the heart tissue section of SHR animals. Representative Western blot depicting the changes in the levels of COL1A1, tPA, and uPA proteins involved in fibrosis in normotensive rats (WKY), spontaneously hypertensive rats (SHRs) and SHRs supplemented with low dose (EJLE_L_) and high dose (EJLE_H_) of EJLE. Results were analyzed by one-way ANOVA using Tukey test with GraphPad Prism software (Version 5.0). # (*p* < 0.05) indicate significant differences when compared to normotensive WKY group (SHR vs. WKY); whereas * (*p* < 0.05) indicate significant differences when compared to SHR group.

Furthermore, as evident from the Figure 6; the levels of collagen type I α 1 chain, COL1A1, the component of ECM was high in the SHR animals. Nonetheless, upon supplementation of EJLE especially in high dosage there was a notable reduction in COL1A1 levels suggesting their anti-fibrotic properties.

We further probed for the tissue-type plasminogen activator proteins including tPA and uPA; consistent with the aforementioned results, we could observe significantly higher levels of these markers in SHR; however, significant reduction in their levels was observed following administration of EJLE particularly in high dose concentration.

**Figure 7:**
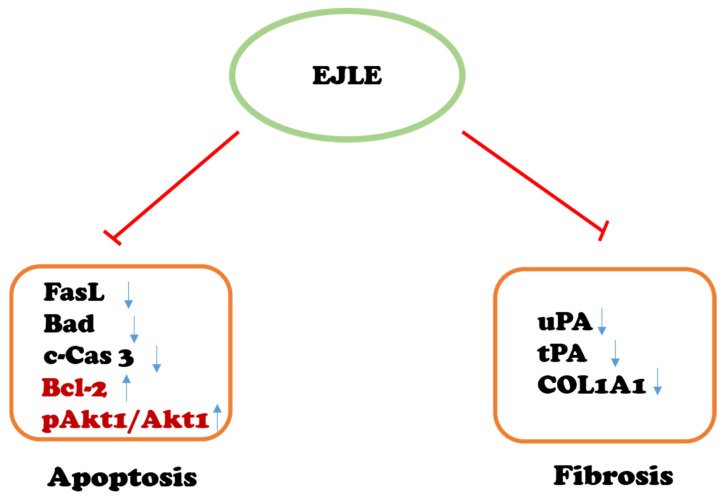
Schematic outline representing the effect of EJLE on cardiac apoptosis mediators (FasL, Bad, c-Cas 3, and anti-apoptotic/survival markers Bcl2 and Akt1) and fibrosis mediators (uPA, tPA, COL1A1) in model animals. Arrow represents proteins modulated by EJLE. The T bar represents attenuation of cardiac apoptosis and fibrosis through supplementation of EJLE, whereas the upward and downward arrows represent the protein expressions pattern.

Collectively, it could be envisaged that EJLE potentially attenuates hypertension-induced cardiac apoptosis and fibrosis; in addition, EJLE could enhance survival (Figure 7). Taken together, attenuation of apoptosis and fibrosis in parallel with enhancement of survival markers could be attributed to amelioration of cardiac functional characteristics. However, it was interesting to note that high and low dose regime significantly rescued crucial cardiac functional characteristics. Nonetheless, a dose-dependent response was observed in attenuation of cardiac apoptosis and fibrosis. Although these intricacies require further investigations, the potential of EJLE to promote cardio-protection certainly widens their horizon to be a plausible strategic intervention against hypertension-induced cardiac ailments.

## 3. Discussion 

In the past decades, hypertensive heart diseases have been one of the leading causes of death [36]. Mounting evidences have shown the cardio-protective effects of various herbal biomedicines against diverse cardiomyopathies. EJ is a popular herbal plant with many pharmacological benefits. Earlier reports have shown that EJLE suppress muscle atrophy in animal models basically by targeting the muscle degradation pathway [37]. Moreover, it embodies potentials to improve myogenic differentiation and function while suppressing muscle loss in an aged rat model [38]. Furthermore, recently, it has been shown to be a natural inhibitor of phosphodiesterase-4D, which is potentially one of the therapeutic targets of inflammatory ailments [39]. Nonetheless, as of yet there is no report highlighting the efficacy of EJLE against hypertension-induced cardiac apoptosis and fibrosis. To the best of our knowledge, this is the first report demonstrating the potential effects of EJLE against cardiac apoptosis and fibrosis in hypertensive SHR animals.

Echocardiography is a multi-purpose non-invasive technique to analyze cardiac function and structure. Accumulating evidences have allocated the importance of amelioration of the cardiac functional characteristics to gauge the cardio-protective attributes of various palliative and curative therapeutic agents [40,41,42]. Echocardiographic analysis clearly suggested that EJLE significantly rescued the crucial cardiac functional parameters viz. EF and FS. Apparently, attenuation of apoptosis and fibrosis in conjunction with enhancement of survival pathways could be attributed to the EJ-mediated rescuing effects of cardiac functional parameters in hypertensive SHRs.

It is of general consensus that cardiac apoptosis and fibrosis are characteristic detrimental factors that plays important role in cardiac remodeling and HF [2]. TUNEL staining and Western blot analysis for apoptosis showed that supplementation of EJLE suppressed apoptosis in SHR animals. The suppression was apparently evident through attenuation of key apoptotic mediators c-Cas3 and other upstream signaling mediators of apoptosis such as FasL and Bad. Furthermore, supplementation of EJLE enhanced the expression of the survival markers i.e., Bcl-2 and pAkt1 in SHRs. Seemingly, it could be envisaged that the protective effects of EJLE against cardiac apoptosis are through attenuation of apoptosis together with enhancement of the survival pathway. Interestingly, these findings concord with reports by the Liu group, wherein they have explicitly demonstrated that suppression of the pro-apoptotic protein Fas and enhancement of the anti-apoptotic protein Bcl-2 play important roles in the attenuation of cardiac apoptosis by allicin in a streptozotocin-induced diabetic rat model [43].

Furthermore, the attenuation of collagen accumulation and fibrosis-related proteins has been widely acknowledged against treatment of fibrosis [44]. Recently Gupta et al., demonstrated that PA inhibitor-1 protected mice against myocardial fibrosis through inhibition of uPA-mediated plasminogen activation [17]. To this end, the considerable reduction of collagen accumulation and fibrosis associated proteins tPA and uPA in SHR animals through supplementation of EJLE potentially supports their efficacy against hypertension-induced fibrosis in model animals.

Cumulatively, as more and more is gleaned about the intricacies underlying cardiac ailments, it could be more instrumental to offer better protection against cardiomyopathies. Nonetheless, the results of the present study certainly instigate that EJLE promotes cardio-protective effects seemingly through amelioration of cardiac apoptosis and fibrosis in hypertensive hearts.

## 4. Materials and Methods

### 4.1. Chemical and Reagent 

All standard reagents were obtained from Sigma-Aldrich (St. Louis, MO, USA) unless and otherwise mentioned.

### 4.2. Preparation of EJLE 

The leaves of the EJ were processed into small pieces and dried under direct sunlight. Subsequent to these, the dried leave pieces (70 gm/1000 mL of MilliQ H_2_O) were thoroughly boiled at 100 °C for stipulated time interval, cooled at room temperature (RT) and filtered. The filtrated were finally collected and stored at −80 °C until further use. For administration of dose, every time, fresh aliquots were prepared.

### 4.3. Animal Model and Experimental Design

All animal experiments were executed in accord with the guidelines for the Care and Use of Laboratory Animals (National Institutes of Health Publication No. 85-23, revised 1996) under a protocol approved by the Animal Research Committee of China Medical University, Taichung, Taiwan. The approval number for the ethical clearance was 2016-065 (08-2016). Twelve-week-old male Wistar Kyoto Rats (WKY) and spontaneously hypertensive rats (SHRs) were in housed in the animal facility center at China Medical University, Taichung, Taiwan under a 12:12 h dark-light cycle, at temperature of about 22 ± 2 °C. All animals have access to food and water ad libitum and after procurement were allowed to acclimatize for at least 2 weeks prior to starting the experiments. At stipulated time intervals, feed intake measurements were taken and health of the animals were recorded. Animals were randomly divided into one of following groups: control WKY rats, SHR rats, SHR rats treated with low dose (100 mg/kg body weight) of EJLE (EJLE_L_) and SHR rats treated with high dose (300 mg/kg body weight) of EJLE (EJLE_H_). Six animals were there in each group. The animals were treated with EJLE through oral gavages twice a week for 8 consecutive weeks. Finally, the animals were euthanized in a prefilled CO_2_ chamber with 100% CO_2_ and sacrificed. The heart tissues were carefully collected for subsequent studies. All necessary procedures were undertaken to reduce pain to the animals.

### 4.4. Echocardiography

Echocardiographic analysis was strictly executed in accord with the guidelines published by the American Society of Echocardiography as described in our previous studies [45,46].

### 4.5. Masson’s Trichrome Staining

The heart tissues were fixed with 10% formalin, treated with series of alcohol gradient (75%, 85%, 90%, and 100% liquor, 5 min each) and embedded in paraffin wax. Paraffin embedded sections were cut into 0.2 μm-thick slices and deparaffinized by submersion in xylene solution. Following deparaffinization, the tissue sections were immersed in preheated Bouin’s fluid for stipulated time, and subsequently rinsed under tap water. Thereafter, the sections were successively stained with Weigert’s iron hematoxylin solution, Biebrich scarlet-acid fucshin, phosphotungstic-phosphomolybdic acid solution, aniline blue solution, and 1% acetic acid solution with intermittent washing procedures. Lastly, the slides were dehydrated in 95% ethanol and finally washed twice with absolute ethanol and xylene and mounted in synthetic resin.

### 4.6. Terminal Deoxynucleotidyl Transferase-Mediated Deoxyuridine Triphosphate (dUTP) Nick End Labeling (TUNEL) and 4',6-Diamidine-2-Phenylindole Dihydrochloride (DAPI) Staining

Cellular apoptosis was assessed using the standard in situ cell death detection kit (Roche Diagnostics, Indianapolis, IN, USA) according to the manufacturer’s instructions as standardized in our lab [47]. In brief, the tissue sections were deparaffinized and rehydrated through series of xylene and ethanol solutions. Permeabilization of tissue was carried out with Proteinase K (Sigma-Aldrich, St. Louis, MO, USA) in 10 mM Tris 7.5 and 5 mM EDTA. Following washing with PBS, the sections were incubated with TUNEL dye at RT for stipulated time interval under dark condition, whereas the nucleus was counter stained with DAPI. Following staining, images were acquired on the fluorescence microscope (Olympus Microscope CKX53, Tokyo, Japan) in a detection range of 515–565 nm.

### 4.7. Tissue Extraction

The LV heart tissue sections were carefully isolated from the whole heart of the animals. For tissue lysate preparation, the LV was homogenized in lysis buffer according to our earlier studies [48]. Following homogenization, the homogenate was clarified by centrifugation at 12,000 rpm for 40 min at 4 °C. The as-obtained tissue lysate was stored at −80 °C until further analysis. The protein concentration was quantified through standard Lowry method [49].

### 4.8. Western Blot

Western blots were performed as described in previous studies [3,20]. Basically, 30–40 μg of the total lysate proteins were resolved through 8–12% SDS-PAGE and transferred to a PVDF membrane (GE Healthcare, Amersham, UK). Nonspecific protein binding was blocked with 5% skimmed milk in Tris-buffered saline-Tween- 20 (TBS-T) for 1h at RT. The membranes were probed with respective primary antibodies Santa Cruz, CA, USA—Akt1 (sc-5298), Bcl-2 (sc-7382), COL1A1 (sc-28657), Fas L (sc-956), Bad (sc-8044), GAPDH (sc-25778), tPA (sc-5239), and uPA (sc-14019); Cell Signaling Technology, Inc, pAkt (#9275), and c-Cas 3 (#9664). All the secondary antibodies (anti-rabbit, mouse and goat, HRP-conjugated) were purchased from Invitrogen (Carlsbad, CA, USA). Subsequent to the usual washing steps, the membranes were thereafter incubated with HRP conjugated secondary antibodies (1:10,000, GE Healthcare, Amersham, UK) and developed with ECL Substrate (Millipore, Billerica, MA, USA) and finally analysed with the LAS-3000 fluorescence imaging system (Fuji Film, Tokyo, Japan).The densitometry analysis of protein expression was performed though Image J software (NIH, Bethesda, MA, USA).

### 4.9. Statistical Analysis

Statistical analysis was performed through GraphPad Prism5 statistical software (San Diego, CA, USA). *p* < 0.05 was considered as significant. Multiple comparisons of the data were analysed through one way analysis of variance (ANOVA) assays. Tukey’s Honestly Significant Difference tests (Tukey HSD) for post hoc comparison were used with a significance level of 5%. All results were quantified employing Image J software and processed through Adobe Photoshop.
